# The Value of Magnetic Resonance Imaging and Endorectal Ultrasound for the Accurate Preoperative T-staging of Rectal Cancer

**DOI:** 10.7759/cureus.30499

**Published:** 2022-10-20

**Authors:** Collins O Opara, Farhana Yaqoob Khan, Dr. Gargi Kabiraj, Humaira Kauser, Jaimee J Palakeel, Mazin Ali, Phani Chaduvula, Sanika Chhabra, Smriti Lamsal Lamichhane, Vaiishnavi Ramesh, Lubna Mohammed

**Affiliations:** 1 Acute Medicine and Geriatrics, California Institute of Behavioral Neurosciences and Psychology, Fairfield, USA; 2 Pathology, California Institute of Behavioral Neurosciences and Psychology, Fairfield, USA; 3 Medicine, California Institute of Behavioral Neurosciences and Psychology, Fairfield, USA; 4 Neurology, California Institute of Behavioral Neurosciences and Psychology, Fairfield, USA; 5 Family Medicine, California Institute of Behavioral Neurosciences and Psychology, Fairfield, USA; 6 Internal Medicine, California Institute of Behavioral Neurosciences and Psychology, Fairfield, USA

**Keywords:** t-staging, staging, endorectal ultrasound, rectal cancer, rectal neoplasm, s: magnetic resonance imaging

## Abstract

Magnetic resonance imaging (MRI) and endorectal ultrasound (ERUS) are essential imaging modalities to assess the depth of tumour invasion (T-staging) in rectal cancer preoperative staging. Accurate T-staging is critical for rectal cancer prognosis and has substantial importance in the determination of appropriate treatment strategies for rectal tumours. There seems to be a knowledge gap in the published literature regarding the most appropriate imaging modality for the preoperative staging of rectal cancer. The purpose of this study was to determine the most appropriate imaging technique for the preoperative T-staging of rectal cancer by comparing the MRI and ERUS staging. In this study, we performed a literature review of studies published in the last 10 years and compared the accuracy, sensitivity, and specificity of ERUS and MRI for the preoperative T-staging of rectal cancer with the aim of identifying the most appropriate imaging modality. The studies reviewed were selected by a rigorous literature search of academic databases. Three electronic databases (PubMed, CINAHL, and Scopus) were searched, and articles were identified. Further rescreening of the articles for those that met the inclusion criteria and searching of the citations of the articles produced eleven journal articles used in this research. Endorectal ultrasound produces accurate results for the T-staging of early rectal cancer, particularly T1 and T2, and has the ability to show the layers of the bowels more clearly in early-stage rectal cancer. However, MRI shows more accurate results for the staging of locally advanced tumours such as advanced T3 and T4 and is particularly important when estimating tumour invasion into the mesorectum, which is very important for the prognostication and survival of patients with rectal cancer. MRI has low accuracy for differentiating early T3 tumours from T1 or T2 with desmoplastic reactions, and therefore, is more likely to overstage these tumours.

## Introduction and background

Colorectal cancer is the second most common cause of death in men after lung cancer [1] and the third leading cause of mortality in women, and the incidence increases after the fourth decade of life [2,3]. Recently, there have been many improvements in the diagnosis and treatment of rectal cancer. Many of these have focused on achieving more accurate preoperative radiological staging [4,5]. The introduction of magnetic resonance imaging (MRI) and endorectal ultrasound (ERUS) has significantly improved the assessment of mural or extramural invasion, which is critical for treatment stratification, especially for those who will benefit from total mesorectal excision (TME) or who require neoadjuvant chemoradiation [6,7].

Tables 1-2 show the T-staging based on the tumour, node, and metastasis (TNM) classification of rectal cancer using magnetic resonance imaging and endorectal ultrasound.

**Table 1:** T-staging of rectal cancer for magnetic resonance imaging T1-T4b: rectal cancer T-staging by magnetic resonance imaging describing extent of tumour invasion through the rectal wall.

T-stage	Rectal wall involvement/description
T1	Limited to the mucosa and submucosa of the rectum
T2	Tumour invasion into the muscularis propria
T3	Tumour invasion into the mesorectum/mesorectal fascia
T4	Tumour invasion into adjacent pelvic structures
T4a	Involvement of the visceral peritoneum or peritoneal reflection
T4b	Tumour invades other pelvic organs

**Table 2:** T-staging of rectal cancer for endorectal ultrasound ERUS: endorectal ultrasound, uT1-uT4: T-staging by endorectal ultrasound describing extent tumour wall invasion through the rectal wall.

T-stage (ERUS)	Rectal Wall involvement/description
uT1	Tumour confined to the submucosa
uT2	Tumour extension into the muscularis propria
uT3	Tumour extension into the mesorectal fat
uT4	Tumour involves adjacent organ or peritoneum

**Figure 1:**
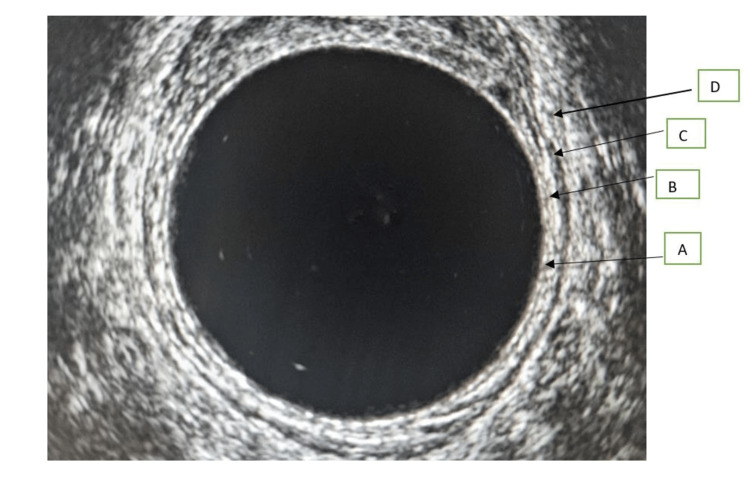
Layers of rectum visualised by endorectal ultrasound A: mucosa, B: submucosa, C: muscularis propria, D: perirectal fat. The black arrows represent the four layers of the rectum as seen on a normal endorectal ultrasound. Created by Collins O. Opara.

The muscularis propria is an important anatomic structure on which the T-staging of rectal cancer is based. The staging by MRI and ERUS is similar except that staging by ERUS has a 'U' before it to differentiate it from MRI staging. T1 tumours are limited to the rectal mucosa and submucosa, and T2 tumours invade the muscularis propria. In the T3 stage, tumour cells invade beyond the muscularis propria into the mesorectum. The T3 stage is further subclassified into three stages based on the tumour distance to the mesorectal fascia (Tables 1-2). Hildebrandt and Feifel were the first to use ERUS in the staging of rectal cancer [8], and this has been accepted as an essential imaging tool for the staging of rectal cancer, especially the T-staging of rectal cancer [9]. The diagnostic accuracy of ERUS, as reported in the literature, is between 80% and 95% [9]. A meta-analysis found the accuracy of ERUS was between 88% and 95%, whereas the sensitivity for staging T1, T2, and T4 tumours was 87.8%, 80.5%, and 95.4%, respectively [10]. The specificity for T1, T2, and T4 tumours was 98.3%, 95.6%, and 98.3%, respectively [10,11]. ERUS, however, is operator-dependent, and its accuracy is highly dependent on the experience and skills of the operator. Three-dimensional ERUS has the capacity for more accurate T-staging compared with conventional ERUS because of its multiplanar imaging capabilities, allowing for better visualisation of the rectal wall [12,13]. Figure 1 shows all the layers of the rectal as seen on conventional endorectal ultrasound imaging.

**Figure 2:**
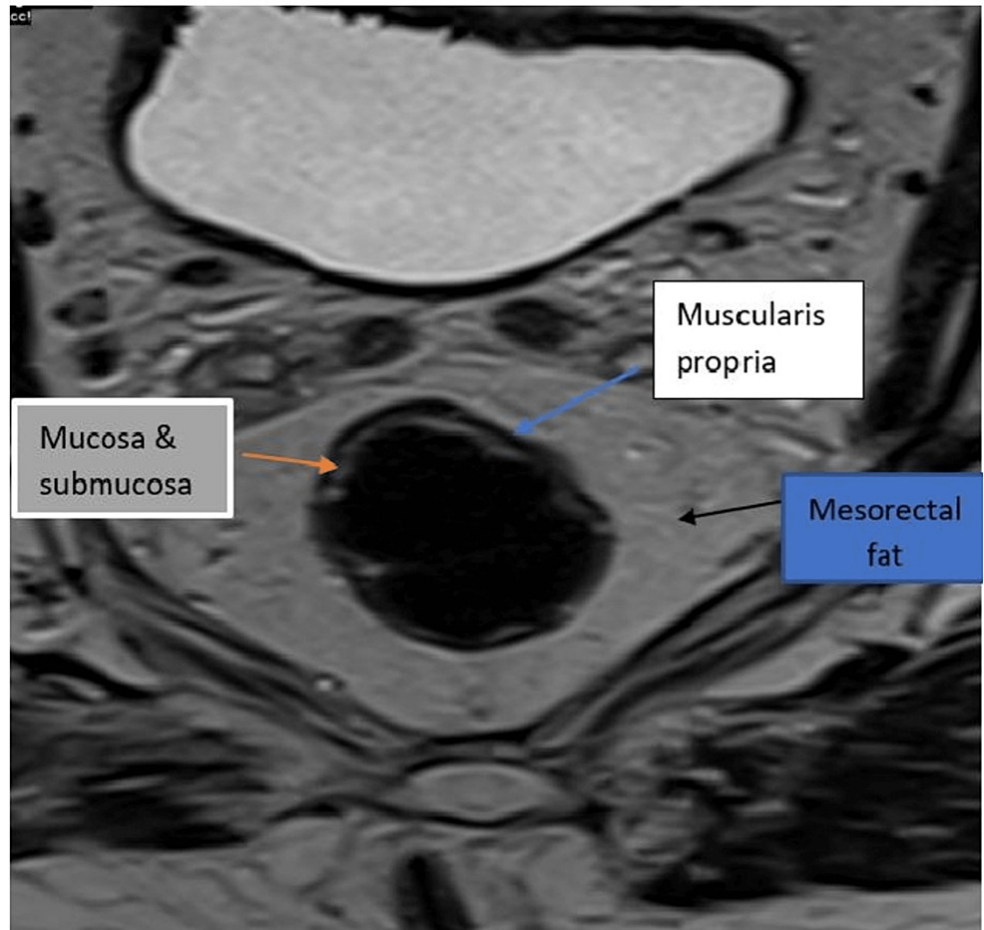
Axial T2-weighted MRI image of the rectum showing the rectal wall and surrounding mesorectum MRI: magnetic resonance imaging. Created by Collins O. Opara.

**Figure 3:**
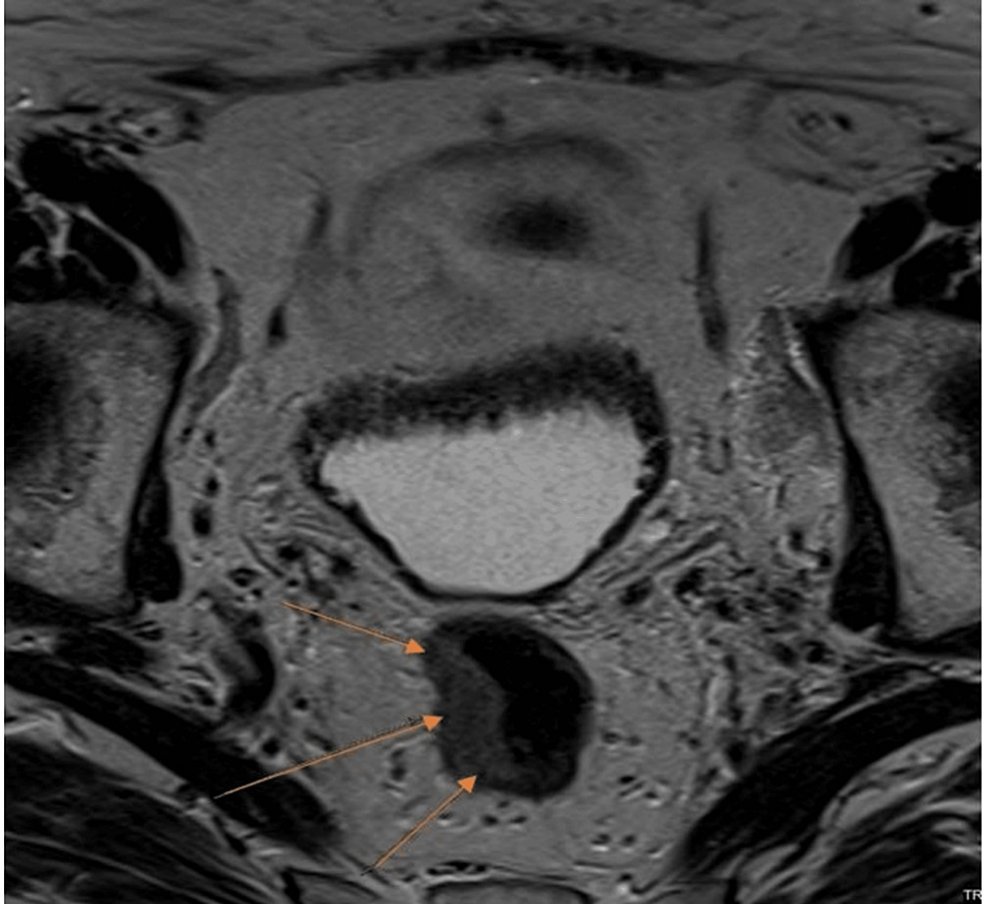
T2-weighted axial MRI image showing a rectal tumour Yellow arrows: rectal tumour, MRI: magnetic resonance imaging. Created by Collins O. Opara.

T2-weighted high-resolution MRI sequences (Figures 2-3) have been shown to be reliable for the assessment of tumour invasion through the rectal wall (T-staging). It is valuable for the measurement of tumour distance from the mesorectal fascia (circumferential resection margin involvement), which is critical for prognostication and treatment selection [14]. Figure 2 shows an axial T2-weighted MRI image showing the rectal wall and the surrounding mesorectal wall, which are all important in the staging of rectal cancer.

Figure 3 is a T2-weighted axial image showing a rectal tumour confined to the rectal wall.

MRI achieves this by providing excellent contrast between normal soft tissue and tumour tissue [1,14]. We performed a literature review to compare the roles of MRI and ERUS in the T-staging of rectal carcinoma and identify the appropriate imaging modality for the T-staging of early or localised advanced disease. This is vital when making a targeted selection of patients for treatment and when identifying those at high risk of local recurrence, which ultimately improves outcomes. In addition, this will provide the surgeon with a roadmap during the surgical treatment.

## Review

Methodology/search strategy

A comprehensive search of published literature was performed for this study. The literature search involved three academic databases (PubMed, CINAHL, and Scopus) searched between March and April 2022 (summarised in Table 3). The scale for assessment for narrative review articles (SANRA checklist) was used for study appraisal. Eligibility for the study included studies that included patients undergoing primary imaging for preoperative staging, studies done in the last ten years, and those published in English. We excluded studies that included patients who underwent chemo-radiotherapy prior to the scans and postoperative patients.

**Table 3:** Summary of search strategy MRI: magnetic resonance imaging, MeSH: medical subject headings.

Databases	Search terms
PubMed	“Rectal neoplasms”[MeSH Terms] OR rectal cancers [Text Word] AND “magnetic resonance imaging" [MeSH Terms] OR MRI scans [Text Word] AND rectal ultrasound OR ultrasound AND preoperative staging.
CINAHL	Magnetic resonance imaging OR MRI AND Rectal cancer OR Rectal neoplasm AND staging* AND endorectal ultrasound.
Scopus	MRI AND endorectal ultrasound AND preoperative staging OR T staging AND rectal cancer.

Results

The sample size used in the journal articles reviewed is between 38 and 234. An overview of the results of the 11 articles reviewed is shown in Table 4. The mean age of the participants was between 54 and 70 years old. The accuracy, sensitivity, and specificity outcomes of the image modalities (MRI or ERUS) in each of the studies are summarised in Table 4. The characteristics of all the relevant articles reviewed are shown in Table 5.

**Table 4:** Summary of results ND: not determined, MWC: modified Wong classification, ESI: external sphincter involvement, ISI: internal sphincter involvement, And: adenoma, DWI: diffusion-weighted image, 3DERUS: 3-dimensional endorectal ultrasound, D1: first ultrasound doctor, D2: second ultrasound doctor, T1-T4(T-staging of rectal cancer by magnetic resonance imaging or endorectal ultrasound), T3ab: T3 substaging, MRI: magnetic resonance imaging, ERUS: endorectal ultrasound, %: percent.

Authors	Sample size (%)	Accuracy (%)	Sensitivity (%)	Specificity (%)	Over staging (%)	Under staging (%)
Surace et al. [15]	77	ND	ERUS		ERUS	ERUS
			T1(77.8)		T1(47.1)	T1(11.8)
			T2(83.3)		T2(33.0)	T2(11.10)
			T3(71.4)		T3(17.7)	T3(23.5)
			T4(83.3)		T4(0)	T4(0)
Kuran et al. [16]	38	Overall=73.7	ERUS	ERUS	ND	ND
		Stenotic group=68	ESI(100)	ESI(96.3)		
		Non stenotic=84.6	ISI(100)	ISI(100)		
		ESI=100				
		ISI=96.8				
Oien et al. [17]	145	ERUS(88)	ERUS(70)	(92)	ND	ND
		MRI(74)	MRI(98)	(16)		
		(Adn, T1 andT2) T3-T4 excluded				
Mondal et al. [18]	53	ERUS	ND	ND	4.1(T1)	6.25
		T1-T2(90%)				
Kolev et al. [19]	71	3DERUS				
		T1(97.1)	T1(92.8)	T1(98.2)	7.6(as T2)	3.4(as T1)
		T2(94.3)	T2(93.1)	T2(95.4)	3.4(as T3)	4.19(as T2)
		T3(95.7)	T3(91.6)	T3(97.8)	Total(2.7)	20(as T3)
		T4(98.5)	T4(100)	T4(98.5)		6.8
		Overall(93)				
Reginelli et al. [20]	97	ERUS	ERUS	ERUS	ERUS	ERUS
		ND	T1(100)	T1(100)	(0)	(0)
			T2(81.8)	T2(95.1)	3	(0)
			T3(95.1)	T3(100)	(0)	13
			T4(90.1)	T4(98.9)	(0)	1.6
			MRI	MRI	MRI	MRI
			T1(66.7)	T1(98.1)	4.5	(0)
			T2(76.2)	T2(94.2)	9.8(as T3)	(0)
			T3(82.1)	T3(87.5)	27	(0)
			T4(90.9)	T4(98.9)	(0)	1.6
					MRI+DWI	MRI+DWI
					T1(0)	(0)
					T2(4.9as T3)	(0)
					T3(0)	(18)
					T4(0)	(0)
Chan et al. [21]	234	ERUS	ERUS	ERUS		
		T1(0.93)	T stage(79)	89		
		T2(0.82)	MRI	MRI		
		T3(0.94)	T stage(79)	85		
		T4(0.76)				
		Total(0.87)				
		MRI				
		T1(0.82)				
		T2(0.77)				
		T3(0.83)				
		T4(0.72)				
		Total(0.82)				
Zhong et al. [22]	61	ERUS(T3)			ND	ND
		D1(86.9)	D1(93)	72.2		
		D2(85.2)	D2(88.4)	77.8		
		MRI(90.2)	MRI(95.3)	77.8		
		T3ab				
		D1(79)				
		D2(64.7)				
		MRI(86.3)				
Munoz et al. [23]	70	ERUS			ERUS(27)	ERUS(4.3)
		T1&T2(68.9)			MRI(22.8)	MRI(4.3)
		T3(58.3)			MWC	
		T4(100)			ERUS(5.7)	11.4
		MRI			MRI(7.1)	2.8
		T1&T2(72.9)				
		T3(63.4)				
		T4(75)				
		MWC				
		ERUS(82.9)				
		MRI(90)				
Patel et al. [24]	75	ERUS(25months)	ERUS(25years)			
		73	70.8	100	26.9	0
		MRI(72.2)	70	100	27.3	0
		12months	12months	12months	12months	12months
		ERUS(78.3)	ERUS(100)	ERUS(100)	ERUS(21.7)	ERUS(0)
		MRI(50)	MRI(0)	MRI(0)	MRI(50)	MRI(0)
Ren et al. [25]	44	ERUS				
		T1(95.5)	T1(85.7)	T1(97.3)	14.3	
		T2(90.9)	T2(87.5)	T2(92.9)	6.3	6.3
		T3(70.5)	T3(88.9)	T3(96.2)		5.9
		T4(97.7)	T4(100)	T4(97.6)		25
		Total(88.6)			Total(4.5)	6.8

**Table 5:** Summary of articles reviewed

Author	Study design	Location	Year of study	Sample size	Mean age (years)
Surace et al. [15]	Retrospective	Italy	2014	77	-
Kuran et al. [16]	Retrospective	Turkey	2014	38	57.6±11.3
Oien et al. [17]	Retrospective	Norway	2019	145	68
Mondal et al. [18]	Prospective	United Kingdom	2014	53	66
Kolev et al. [19]	Retrospective	Bulgaria	2014	71	61.3±12.2
Reginelli et al. [20]	Retrospective	Italy	2021	97	66
Chan et al. [21]	Meta-analysis	Canada	2019	234	-
Zhong et al. [22]	Retrospective	China	2017	61	54.36±10.93
Munoz et al. [23]	Retrospective	Spain	2013	70	70.4
Patel et al. [24]	Retrospective	United Kingdom	2014	75	69
Ren et al. [25]	Retrospective	China	2012	44	63.3±10.2

Discussion

Extensive studies have investigated the role of MRI and ERUS in the preoperative T-staging of rectal cancer. In this section, we will examine the results of the relevant literature identified and reviewed. Surace et al. observed that the diagnostic sensitivity of ERUS for T-staging was quite significant across all the T-stages, consistent with previous studies [10,11,15]. However, the sensitivity of ERUS for T3 tumours was the lowest (71%) among the T-stage subgroups [15]. This might be due to its poor ability to characterise tumour extension beyond the muscularis propria into the mesorectum [1]. In terms of specificity, ERUS showed significant specificity for the T-staging of rectal cancers. Furthermore, T1 and T2 tumours had a greater propensity to be overstaged with ERUS compared with other T stages [15]. This might be due to the habit of overstaging a suspicious tumour to prevent the undertreatment of patients. In addition, T3 tumours showed the most significant risk of being understaged (23.5%) [15], which can result in inappropriate categorisation, ultimately leading to undertreatment. This understaging risk was most likely due to interpretation bias and difficulty in assessing tumours located higher up the rectum related to difficulties with the ultrasound probe when evaluating these tumours [15]. Overall, ERUS was an important radiological modality for the preoperative T-staging of T1 and T2 tumours and assisted in deciding or selecting patients qualified for surgery.

Kuran et al. studied the staging of low rectal cancer using ERUS [16]. In that study, ERUS was used to assess the T-staging of low rectal tumours in two categories: those with or without stenotic lesions. They recorded an overall accuracy of 73.7%, which was consistent with 65-97% recorded in previous studies [17,18]. However, ERUS showed less accuracy for the staging of stenotic tumours (68%) compared with non-stenotic lesions (84.6%), although the results obtained were still within acceptable limits [16]. Although this disparity can be reduced by using a mini probe during the examination, this is still not common practice, especially in developing countries. This exposes one of the shortcomings of ERUS for the assessment of rectal tumours and further emphasises the importance of a multi-imaging approach in the preoperative assessment of rectal cancers. Despite these shortcomings, its high sensitivity and specificity for the assessment of external sphincter involvement (ESI) and internal sphincter involvement in low rectal tumours showed that ERUS remains invaluable for the assessment of low rectal cancer [16] (Table 4).

Oien et al. compared the accuracy of ERUS and MRI for staging and distinguishing rectal adenomas from early-stage rectal cancer [17]. ERUS showed high accuracy in the T-staging of rectal cancer (88%) [17], including the accurate identification of adenoma compared to MRI (75%) [17]. It is relevant to note that the study excluded T3 and T4 tumours, which might have affected the reliability of the results obtained as only early stage tumours and adenomas were assessed. Despite this limitation and consistent with previous studies, ERUS was shown to be much better than MRI for the accurate identification of adenomas and early disease (T1 and T2). However, MRI overstaged most adenomas as T1 and T2 tumours [17]. In situations where an adenoma is suspected by rectal examination or colonoscopy, MRI seems to not have any significant benefit for the preoperative staging of adenomas [17]; therefore, using MRI in such situations is cost-effective, especially in poor resource countries.

Kolev et al. reported their experience with the use of the three-dimensional mode (3D) for the evaluation of tumour invasion into the rectal wall. Three-dimensional ultrasound is a recent advancement of conventional two-dimensional ultrasound, which produces a high-resolution image visualised in multiple planes [19]. Three-dimensional ERUS significantly increased the overall accuracy (92.9%) [19] compared with the use of the conventional ultrasound mode [15-19]. The sensitivity and specificity were significantly improved compared with results obtained by conventional ERUS [15-17] (Table 4). The results obtained indicate that 3D ultrasound has the capacity to correctly differentiate early stage rectal disease from advanced stages more than conventional ERUS. Similar to previous studies reviewed [15], more T1 disease was overstaged. However, more T4 tumours were understaged, in contrast to a study reported by Surace et al. [15]. In total, about 2.75% of cases were overstaged [19], whereas only 6.75% [19] were understaged, lower than that recorded in other studies [15].

Reginelli et al. used a multimodal technique, which involved the combination of MRI, MRI with diffusion-weighted imaging (DWI), and ERUS for the assessment of T-staging [20]. The T-staging was initially assessed with ERUS, high-resolution T2-weighted MRI, and a combination of DWI with T2-weighted MRI, separately [20]. MRI alone showed a sensitivity rate of 66.7-90.9% and a specificity of 87.5-98.9% [20], which was consistent with the previous literature [1,26-29]. However, it was less sensitive for the identification of T1 and T2 tumours when compared to the other T subgroups leading to the overstaging of early rectal cancers, with about 9.8% of T2 tumours staged as T3 (Table 4). This further validates the already established challenge documented in the literature [30] regarding the diagnostic challenge associated with the use of MRI for the staging of early rectal disease. The reason for this has been attributed to the difficulty of MRI in differentiating tumour infiltration into the mesorectal fat and a desmoplastic tissue reaction, which results in the overstaging of T2 and early T3 tumours by MRI [31-36]. Interestingly, a new finding reported by Reginelli et al. research showed that the combination of DWI and T2-weighted MRI resulted in an overwhelming increase in the diagnostic sensitivity (100%), specificity (100%), and positive predictive value (100%) of the subgroups of T-staging [21]. This was quite significant when compared to the results obtained by MRI or ERUS (Table 4). This resulted in the reduction of overstaged T2 tumours staged as T3 to 4.9% [20]. This finding demonstrated the higher capability of DWI to delineate tumours from fibrotic or inflammatory reactions around tumours resulting in higher sensitivity for T2 and T3 tumours, thereby reducing the risk of staging failure [21,30-31]. A multiparametric construction of the images, which is a combination of all the imaging modalities, obtained from the three techniques studied, resulted in higher sensitivity and specificity for all the T-stage categories compared with the previous techniques described [20]. However, ERUS, demonstrated significant diagnostic performance in terms of sensitivity, specificity, and positive predictive value, especially for early-stage tumours [20]. This was consistent with previous findings discussed; however, ERUS had a higher sensitivity for T3 disease than conventional T2-weighted MRI preoperative staging [21]. Furthermore, a systematic review by Chan et al. reported that ERUS exceeded MRI in the overall T-staging of rectal cancer, in particular T1 and T3 tumours, consistent with other studies [19,20,21]. However, MRI was better for the detection of T2 tumours, a slight deviation from what is already known.

Zhong et al. specifically assessed the roles of MRI and ERUS for the staging and sub-staging of T3 rectal cancer by measuring the extent of mesorectal invasion (EMI) [22], which is an important prognostic factor for the management of rectal cancer and guides surgical decisions [21,22]. The accuracy was assessed by comparing the MRI or ultrasound staging with the histological specimen results. Two sonographers independently assessed the EMI on ultrasound, while a radiologist measured the EMI on MRI. The overall accuracy for T-staging for the two sonographers was similar, at 85.2 and 86.9%, respectively, whereas the overall accuracy of MRI was 80.3% [22]. MRI was superior to ERUS for the staging and sub-staging of T3 tumours [22], whereas MRI was more reliable than ERUS for the measurement of EMI, as documented in the literature [33].

Munoz et al. assessed the use of Wong's classification for the T-staging of rectal cancer and found it improved the accuracy of MRI or ERUS when assessing the depth of tumour invasion in the rectal wall compared with the standard TNM staging [23]. Nardone et al. [35] classified the T3 staging into superficial groups and deep groups to allow for the easy selection of patients for TME or chemoradiotherapy (CRT) [35]. Using ERUS, T3 tumours were subclassified into T3a and T3b using a limit of 19 mm tumour thickness [36]. Using MRI, T3 tumours were sub-grouped into T3a (with mesorectal invasion ≤5 mm) and T3b (mesorectal invasion ≥5 mm) [37]. With the use of the standard TNM classification, the overall accuracy achieved with ERUS was 68.6%, whereas MRI achieved an accuracy of 72.9%. Using the modified Wong classification, there was a substantial improvement in the overall accuracy of ERUS to 82.9% and MRI to 90%. The mean accuracy of both imaging techniques improved from 70.7% to 86.5% [23]. This suggests the use of the Wong classification could improve the overall accuracy of MRI and ERUS for preoperative staging and can improve the already known challenges associated with the MRI staging of T3 tumours. This might enhance patient selection for TME or neoadjuvant RCT; however, more research is required to validate its use and application.

Patel et al. assessed whether the learning curve for the use of ERUS improved the accuracy of ERUS in the T-staging of early rectal cancer (confined to the mucosa and submucosa) because the results obtained from ultrasound are largely operator-dependent [24]. This was initially carried out retrospectively for an initial 25-year period and subsequently repeated after a 12-year period to determine whether the learning curve improved the accuracy of the subsequent T-staging [24]. Over the two periods studied, an increase in accuracy from 73.1% to 78.3%, an increase in sensitivity from 70.8% to 77.35, and a decrease in overstaging from 26.9% to 21.75 were recorded [24]. The results obtained agreed with results from studies that noted that the learning curve over time improved the accuracy of ERUS for the T-staging of rectal cancer [38-42]. Ren et al. reported that ERUS T-staging agreed with the pathological results in 39 out of 44 patients examined, which is equivalent to 88.6% [25]. These results are comparable to the results of other studies reviewed and those documented in the published literature [43-44]. About 4.5% of the tumours were overestimated, and 6.8% of the total cases were understaged [25]. Patients with T1 tumours were overstaged more often compared with T2 tumours (6.3%) [25]. T1, T2, and T4 tumours were staged more accurately than T3 tumours [25]. The presence of microscopic invasion beyond the rectal layers, which was difficult to identify during radiological staging but was seen by histopathological examination, is likely responsible for the understaged cases. The specificity obtained was high and similar across the T subgroups. The high negative predictive value seen in this study across all the T-staging further established what is already discussed in the literature regarding the reasonable accuracy of ERUS to distinguish between early and advanced rectal cancer correctly [15,19,20,45].

Limitations

Most of the literature reviewed the use of ERUS, with few studies evaluating the role of MRI in T-staging. This might have skewed the findings of this study in favour of ERUS. In addition, only 11 studies were reviewed, which is small and therefore cannot be used for generalisation; however, this might be due to the limitations of studies reported in the last ten years. Therefore, more studies that review a larger number of articles will be needed in the future to provide a robust evaluation of both imaging modalities. Finally, all the studies reviewed were conducted in developed countries, probably because of the paucity of studies from developing countries. This might have an important effect on the comparison of experiences in both regions for the purpose of achieving robust results. This should be explored in future studies.

## Conclusions

ERUS is a reliable imaging modality for the assessment of tumour invasion into the rectal wall (T-staging). However, its accuracy is largely dependent on the experience and skills of the operator. The diagnostic accuracy of ERUS is high for early rectal tumours (T1 and T2); therefore, it is more efficient for the staging of early disease. Although its accuracy for late disease (T3 and T4) is low, as reported in our research, the results were still within acceptable limits. ERUS is limited in the staging of stenotic tumours and tends to overstage T2 tumours with surrounding inflammation or fibrosis as it cannot accurately differentiate between tumour tissues and peritumoral fibrosis. Three-dimensional ultrasound has shown promising results in reducing the staging failure seen with conventional ERUS. In experienced hands, it has shown more accurate results; however, more studies are required to validate this. In contrast, MRI is more effective and reliable for the staging of advanced disease (T3 and T4) with much better accuracy for the assessment of tumour invasion into the mesorectal fat or fascia and estimation of the circumferential resection margin, which is a critical factor when selecting patients for different treatment strategies. However, staging failure has been reported in the differentiation of early T2 tumours with peritumoral inflammation from T3 tumours as a result of its poor ability to delineate tumour tissues from inflammatory tissues. Therefore, these tumours tend to be overstaged with the risk of overtreatment.

Finally, MRI and ERUS are essential imaging tools for the T-staging of rectal cancer, each with their own strengths and limitations. However, both should be used as complementary imaging tools for the T-staging to provide an accurate preoperative evaluation of patients with rectal cancer, which will ultimately improve patient outcomes and reduce the risk of local recurrence.
